# Classification of Parkinson’s disease with freezing of gait based on 360° turning analysis using 36 kinematic features

**DOI:** 10.1186/s12984-021-00975-4

**Published:** 2021-12-20

**Authors:** Hwayoung Park, Sungtae Shin, Changhong Youm, Sang-Myung Cheon, Myeounggon Lee, Byungjoo Noh

**Affiliations:** 1 Department of Health Sciences, The Graduate School of Dong-A University, Saha-gu, Busan, Republic of Korea; 2 Department of Mechanical Engineering, College of Engineering, Dong-A University, Saha-gu, Busan, Republic of Korea; 3 Department of Healthcare and Science, College of Health Sciences, Dong-A University, 37 Nakdong‑Daero, 550 Beon‑gil, Hadan 2-dong, Saha-gu, Busan, 49315 Republic of Korea; 4 Department of Neurology, School of Medicine, Dong-A University, 26, Daesingongwon-ro, Seo-gu, Busan, 49201 Republic of Korea; 5 Department of Health and Human Performance, Center for Neuromotor and Biomechanics Research, University of Houston, Houston, TX USA; 6 Department of Kinesiology, Jeju National University, Jeju-si, Jeju-do Republic of Korea

**Keywords:** Parkinson’s disease, Machine learning, Turning, Falls, Kinematics

## Abstract

**Background:**

Freezing of gait (FOG) is a sensitive problem, which is caused by motor control deficits and requires greater attention during postural transitions such as turning in people with Parkinson’s disease (PD). However, the turning characteristics have not yet been extensively investigated to distinguish between people with PD with and without FOG (freezers and non-freezers) based on full-body kinematic analysis during the turning task. The objectives of this study were to identify the machine learning model that best classifies people with PD and freezers and reveal the associations between clinical characteristics and turning features based on feature selection through stepwise regression.

**Methods:**

The study recruited 77 people with PD (31 freezers and 46 non-freezers) and 34 age-matched older adults. The 360° turning task was performed at the preferred speed for the inner step of the more affected limb. All experiments on the people with PD were performed in the “Off” state of medication. The full-body kinematic features during the turning task were extracted using the three-dimensional motion capture system. These features were selected via stepwise regression.

**Results:**

In feature selection through stepwise regression, five and six features were identified to distinguish between people with PD and controls and between freezers and non-freezers (PD and FOG classification problem), respectively. The machine learning model accuracies revealed that the random forest (RF) model had 98.1% accuracy when using all turning features and 98.0% accuracy when using the five features selected for PD classification. In addition, RF and logistic regression showed accuracies of 79.4% when using all turning features and 72.9% when using the six selected features for FOG classification.

**Conclusion:**

We suggest that our study leads to understanding of the turning characteristics of people with PD and freezers during the 360° turning task for the inner step of the more affected limb and may help improve the objective classification and clinical assessment by disease progression using turning features.

**Supplementary Information:**

The online version contains supplementary material available at 10.1186/s12984-021-00975-4.

## Background

Freezing of gait (FOG) has been defined as an episodic inability to generate effective forward stepping movements in the absence of any known cause other than Parkinsonism or high-level gait disorders [1]. The FOG symptom is commonly observed throughout the progression of Parkinson’s disease (PD); it is a significant risk factor for falls and contributes to functional incapacity, thus reducing the quality of life [2–4]. FOG in people with PD is a sensitive problem, which is caused by motor control deficits and requires greater attention during postural transitions such as turning [5, 6] or in challenging situations (e.g., passing through narrow passages or crowded spaces, dual tasking, etc.) [2]. Especially, turning is impaired during disease progression owing to the asymmetric characteristics of the turning phase and asymmetric symptom distribution in people with PD [7–9]. Although not all people with PD develop FOG, it may appear with disease progression as is evidenced by the continued manifestation of FOG in the “On” state of medication and its relationship with other levodopa resistant symptoms such as postural instability [5, 10]. Therefore, the turning characteristics of people with PD have been researched to improve the evaluation of disease prognosis and classify people with PD with and without FOG (freezers and non-freezers).

Previous studies on the turning characteristics in freezers have reported increased turn duration, cadence, number of steps, decreased peak turn velocity [6], decreased bilateral coordination [11], increased temporal and spatial variability [2, 12], reduced medial deviation, forward shift of the center of mass (COM), and decreased step width [7, 13] when compared with non-freezers. However, these studies reported limitations such as small sample size, analysis of only the start or end of the turning phase, turning in the preferred direction, or attaching one or few inertial sensors on the trunk, lumbar, or foot; these approaches may not detect some of the variables that are most sensitive to disease progression [6, 14]. In addition, previous studies reported that people with PD might also be influenced by disease asymmetry owing to spontaneous turning toward the less affected side, thus keeping the more affected limb at the outer side during turning [15, 16]. However, the results of the turning characteristics related to disease asymmetry or unilateral symptoms reported in the previous studies do not include this aspect. Therefore, we aimed to analyze the turning characteristics of the more affected limb in freezers and non-freezers by using full-body kinematic measures.

In addition, some studies were recently conducted to evaluate the optimal combination of turning characteristics for improving the classification and prediction performance of freezers among people with PD [12, 17, 18]. These studies have suggested that the occurrence of FOG while turning is associated with structures [12] such as the prefrontal areas, central pattern generators in the spinal cord, mesencephalic locomotor region, and executive frontal regions [19, 20]. Modeling using machine learning algorithms based on data combined with comprehensive turning characteristics was recently conducted [21–23]. Previous studies have identified the predictors for classification of people with PD as faller and non-faller [20]; these have determined the optimal combination of turning characteristics to distinguish between people with PD and controls [17, 25] as well as categorized people with PD as freezers and non-freezers based on the classifiers [18]. The studies reported a classification accuracy of approximately 70–98% when logistic regression (LR), random forest (RF), support vector machine (SVM), extreme gradient boosting [24], probabilistic neural network [18], recursive feature elimination technique with SVM [25], and partial least square discriminant analysis [17] were used for training. However, these machine learning studies were limited to small sample sizes, analyzed limited turning characteristics, and were vulnerable to the risk of overfitting the data owing to high correlation with multiple variables [26]. In addition, precise classification may not be possible as the models in these studies could not predict specific clinical outcomes or diagnostics according to disease progression based on comprehensive and in-depth turning characteristics due to the use of movement data from only wearable sensors in people with PD or freezers. Thus, the turning characteristics related to movement control and coordination during turning tasks must be categorized to improve the classification performance of freezers and non-freezers.

Therefore, the aim of this study was (i) to evaluate the accuracy of machine learning models with feature selection by stepwise regression based on 360° turning characteristics for resolving the classification problem between people with PD and controls and between freezers and non-freezers, and (ii) to investigate the associations between the clinical characteristics and turning features based on the 360° turning features that best classify people with PD and freezers, selected by stepwise regression. We hypothesize that the 360° turning features based on full-body kinematic analysis will demonstrate the objective classification accuracy and associations with clinical characteristics owing to differences between turning characteristics of people with PD and freezers when compared with those of controls and non-freezers, respectively.

## Methods

### Participants

**Fig. 1:**
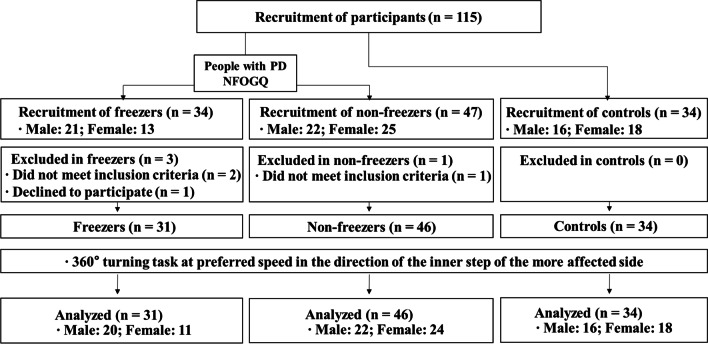
Study flow chart

A total of 77 people with PD (31 freezers and 46 non-freezers) and 34 age-matched older adults as controls participated in this study. A flow chart of the details of the study participants is shown in Fig. 1, and the physical and clinical characteristics of the participants are shown in Table 1. The people with PD were diagnosed by a neurology specialist based on the UK Parkinson’s Disease Society Brain Bank criteria [27]. The inclusion criteria were as follows: (a) aged 55–85 years, (b) who could walk and move independently, with a modified Hoehn and Yahr stage of 2–3 [28, 29], (c) Mini Mental State Examination (MMSE) score > 24 [30], (d) who stably responded to antiparkinsonian medications, and (e) were classified as freezers and non-freezers, i.e., assessed as with and without FOG owing to a score of > 3 and ≤ 3 on the New Freezing of Gait Questionnaire (NFOGQ), respectively [31]. Only one participant classified as a freezer froze during the turning task. This participant was excluded from the analysis. Participants with a history of cardiovascular, musculoskeletal, vestibular, or other neurological diseases, patients who required assistive devices for moving, and patients with dyskinesia that was uncontrollable with drug therapy were excluded. The controls included healthy individuals with no medical history related to cognitive impairment and gait disturbance in the past 6 months and no history of orthopedic surgery.

**Table 1:** Physical and clinical characteristics of all participants

	People with PD		Controls (n = 34)	p-value
	Freezers (n = 31)	Non-freezers (n = 46)		
Sex (male/female)	20/11	22/24	16/18	0.283^a^
Age (years)	69.10 ± 5.29	70.40 ± 5.60	68.82 ± 5.82	0.402^b^
Height (cm)	159.46 ± 9.37	156.61 ± 8.62	159.40 ± 6.80	0.132^c^
Body weight (kg)	60.59 ± 8.47	59.78 ± 8.11	61.62 ± 7.15	0.593^b^
BMI (kg/m^2^)	23.80 ± 2.45	24.38 ± 2.72	24.24 ± 2.25	0.605^b^
MMSE (scores)	27.84 ± 1.97	26.78 ± 2.02	27.56 ± 2.08	**0.026**^c^
Disease duration (years)	8.39 ± 5.83	4.36 ± 3.61	–	**< 0.001**^d^
Treatment duration (years)	7.62 ± 6.39	3.61 ± 3.56	–	**0.001**^d^
L-Dopa equivalent dose (mg/day)	718.23 ± 332.75	468.59 ± 213.99	–	**< 0.001**^d^
NFOGQ (scores)	12.32 ± 7.30	–	–	–
Hoehn and Yahr scale	2.61 ± 0.42	2.37 ± 0.43	–	**0.017**^d^
UPDRS total (scores)	53.37 ± 14.52	45.59 ± 11.27	–	**0.010**^e^
UPDRS part III (scores)	35.00 ± 8.92	33.90 ± 7.63	–	0.565^e^
UPDRS-PIGD (scores)	1.13 ± 0.48	0.54 ± 0.31	–	**< 0.001**^d^
More affected limb (left/right)	20/11	27/19	All right-handed	**< 0.001**^a^

All data are represented as mean ± standard deviations

*PD* Parkinson’s disease, *BMI* body mass index, *MMSE* mini mental state examination, *L-dopa* Levodopa, *NFOGQ* new freezing of gait questionnaire, *UPDRS* Unified Parkinson’s Disease Rating Scale, *PIGD* postural instability/gait difficulty

Boldface denotes a significant difference between groups (p < 0.05)

^a^Fisher’s exact Test

^b^One-way ANOVA

^c^p-value of Kruskal–Wallis *H* Test

^d^Mann–Whitney *U* Test

^e^Independent samples *t*-test

All experiments were performed in accordance with the relevant guidelines and regulations. The experimental protocols were approved by the Institutional Review Board (IRB) of Dong-A University Medical Center (IRB number: DAUHIRB-17-033), and all participants signed a written informed consent before participating in this study.

### Test procedures

All measurements were assessed in the “Off” antiparkinsonian medication state, with medication withdrawn at least 12 h prior to the measurements. The experiments were divided into two sessions. In the first session, the participants completed the informed consent form and were assessed using the Unified Parkinson’s Disease Rating Scale (UPDRS) [32], modified Hoehn and Yahr scale, NFOGQ, and MMSE (Table 1). In the second session, all participants warmed up and practiced turning before the experiment started. Then, the participants were instructed to practice the 360° turning task at a self-selected preferred speed with 3 to 5 trials, and the measurements were conducted after approximately 5 min of rest. Thus, the participants successfully performed the 360° turning tasks three times, with 30 s of rest between trials.

**Fig. 2:**
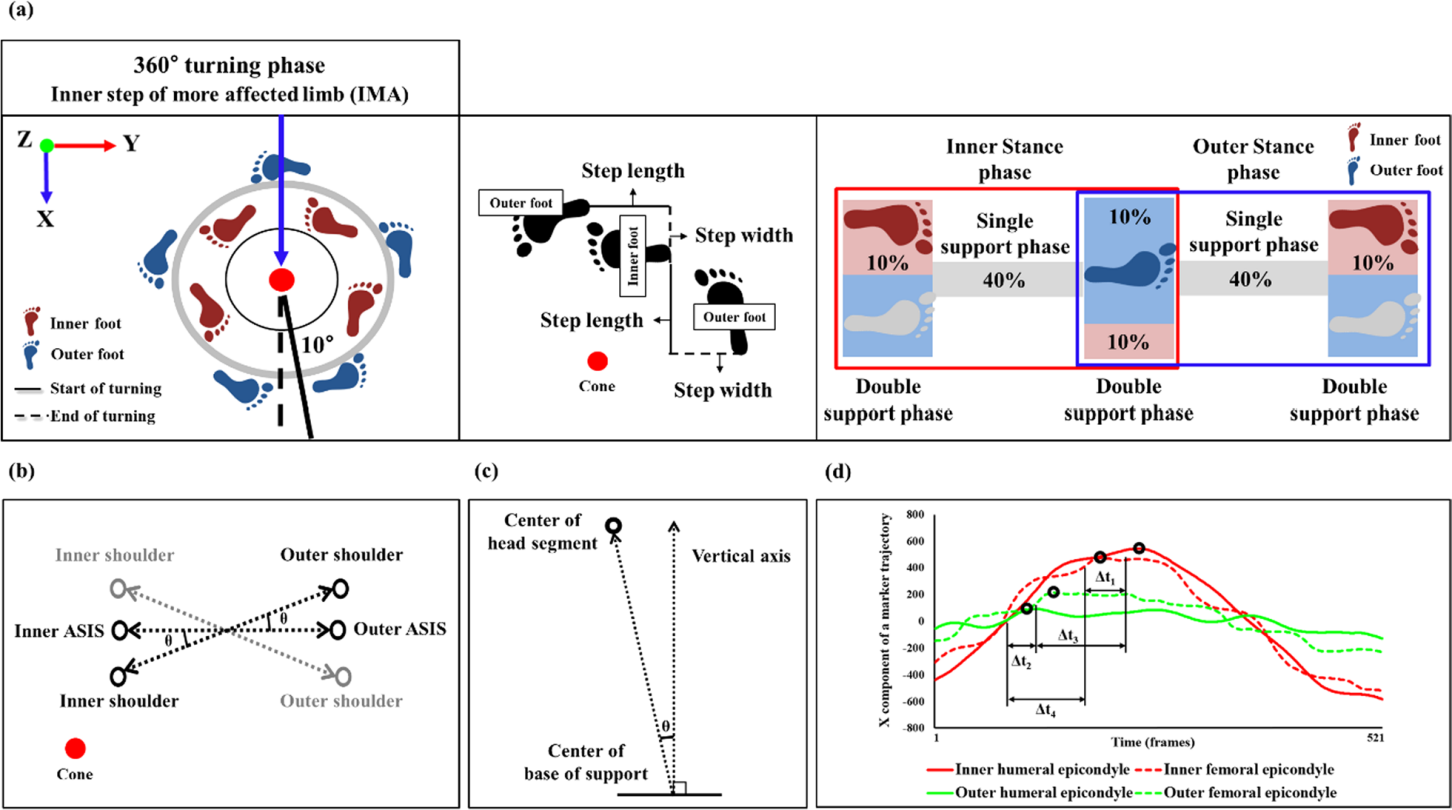
Schematic of experimental setup and analysis phase. (**a**) The 360° turning analysis phase for the inner steps of the more affected limb, the definition of step width, inner and outer step lengths, inner and outer single and double support, and stance phases; (**b**) the definition of the maximum anti-phase; (**c**) the definition of the incline angle; (**d**) the definition of the inner and outer ipsilateral and contralateral temporal coordination parameters of the upper and lower limbs. *ASIS* anterior and posterior superior iliac spine (Fig. 3)

The more affected limb performed the inner steps of the 360° turning tasks (Fig. 2a). The participants were asked to turn comfortably at the preferred speed around a cone. It has been reported that freezers experience difficulty in turning toward the more affected limb [9, 13, 16]. The direction of the more affected limb was defined as the side of disease dominance, which was determined for each PD patient based on the difference between the left and right scores on items 20–26 and item 31 of the UPDRS during the “Off” state of medication, assigned after examination by a neurologist [33].

**Fig. 3:**
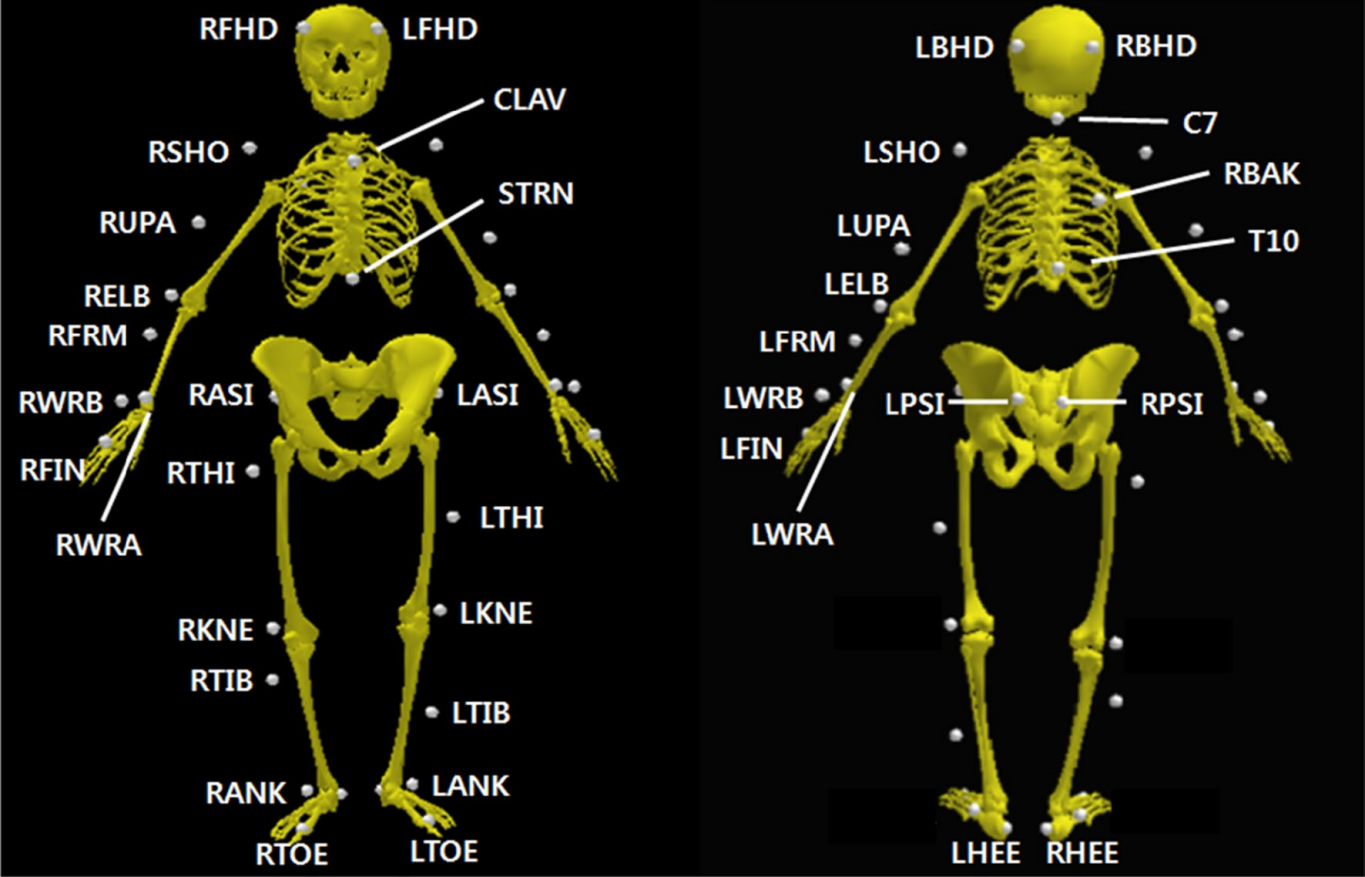
Placement of thirty-nine reflective markers and the corresponding anatomical landmarks. The markers were attached on the clavicle (CLAV), sternum (STRN), 7th cervical vertebrae (C7), 10th thoracic vertebrae (T10), scapular medial border (RBAK), bilaterally on the front and back of the head (LFHD, RFHD, LBHD, and RBHD), shoulder (LSHO and RSHO), lower third of the upper arm (LUPA and RUPA), lateral humeral epicondyle (LELB and RELB), lower third of the forearm (LFRM and RFRM), mediolateral styloid processes of the wrist (LWRA, RWRA, LWRB, and RWRB), third metacarpal head (LFIN and RFIN), anterior and posterior superior iliac spine (LASI, RASI, LPSI, and RPSI), lower third of the lateral thigh (LTHI and RTHI), lateral femoral epicondyle (LKNE and RKNE), lower third of the lateral shank (LTIB and RTIB), calcaneus (LHEE and RHEE), lateral malleolus (LANK and RANK), and second metatarsal head (LTOE and RTOE)

Six infrared cameras (Vicon MX-T10, Oxford Metrics, UK) were used in the three-dimensional (3D) motion capture system. The sampling frequency for the data was 100 Hz. A global coordinate system was established, with the positive X-axis to the right, the positive Y-axis facing anteriorly, and the Z-axis defined as the cross-product between the X-axis and Y-axis, with the positive Z-axis facing superiorly (Fig. 2a). Height, body weight, shoulder offset, elbow width, wrist width, hand thickness, leg length, knee width, and ankle width measurements were obtained; the appropriate metrics were measured bilaterally to estimate the joint kinematics data. The placement of thirty-nine reflective markers in the shape of 14 mm spheres was performed according to the Plug-in Gait full body model (Vicon Motion Systems Ltd., Oxford Metrics, UK), a modified version of the Helen Hayes marker set [34] (Fig. 3).

### Data analysis

The 3D motion analysis data were collected and analyzed using the Nexus software (version 2.10.3, Vicon, UK) and MATLAB R2017b (MathWorks, Natick, MA). The collected data were filtered using a fourth-order Butterworth low-pass filter with a 10 Hz cut-off frequency through frequency analysis. The measurements were obtained three times, and the averaged value was used for the analysis. The start event of the analysis phase during the 360° turning task was determined as the instant when the angle between the pelvic vector (defined as the distance between the left ASIS and right ASIS) and the ML vector (defined as the X-axis vector of the global coordinate system) passed 10°, whereas the event when the two vectors completed 360° was defined as the end of the rotation. Therefore, the analysis was performed through the 350° turning phase [13] (Fig. 2a).

The following spatiotemporal variables were analyzed (Fig. 2): (1) the total number of steps and duration of the turning phase, step width, inner and outer step lengths, inner and outer single and double support, and stance phases. The step width was defined as the length between the initial foot heel contact of one limb and that of the other limb. The inner and outer step lengths were defined as the perpendicular distances between the initial foot heel contact of the inner/outer limb and the initial foot heel contact of the other limb, respectively. The period during which both the inner/outer feet were in contact with the ground during the turning was defined as the inner and outer double support phase. The period during which only the inner or outer foot was in contact with the ground was defined as the inner or outer single support phase, respectively. The inner/outer stance phase was defined as the phase during which the inner/outer foot was in contact with the ground, moving from heel strike to toe off (Fig. 2a). (2) With regard to the kinematic variables, the ROM was calculated as the difference between the maximum and minimum joint (inner and outer hip, knee, ankle, shoulder, pelvic, and thorax) angles on the sagittal plane during 360° turning. The inner and outer toe clearance heights were calculated as the maximum vertical height of the toe marker during the swing phase of each step. In addition, the maximum anti-phase was calculated as the maximum angle (θ) between the pelvic vector from the inner to outer marker of the ASIS and shoulder vector from the inner to outer marker of the shoulder in the horizontal plane during 360° turning (Fig. 2b). The incline angle was calculated as the maximum angle (θ) between the vector from the center of the base of support (calculated as the point of the axis vector connecting the body centroid (center of mass) and the base of support reference point) to the center of the head segment (calculated as the centroid of the 4 head markers) and the vertical axis of the cone during 360° turning (Fig. 2c). The inner and outer ipsilateral and contralateral temporal coordination parameters of the upper and lower limbs were calculated as the temporal differences (Δt) between the peaks of the lateral humeral epicondyle and lateral femoral epicondyle markers on the inner–inner (Δt_1_)/outer–outer (Δt_2_) (ipsilateral) and inner–outer (Δt_3_)/outer–inner (Δt_4_) (contralateral) limbs (Fig. 2d). (3) The area of 95% confidence interval (CI), AP and ML root mean square (RMS) distances, and total distance and velocity of the COM on the horizontal plane during the 360° turning tasks were calculated [13, 35]. The variables analyzed during the 360° turning task are summarized in Additional file 1: Table S1, including the means, standard deviations (SD), p-values, and Cohen’s d effect sizes.

### Statistical analysis

Data normality was checked using the Shapiro–Wilk test. A one-way analysis of variance (ANOVA) and independent t-test or nonparametric statistics were applied to analyze the mean and SD of the physical and clinical characteristics of all participants.

Univariable and multivariable logistic regression analyses using stepwise regression were performed to identify the best combination of the turning characteristics for the optimal classification of people with PD and controls, and freezers and non-freezers. Variables that were significant at p < 0.05 were examined for multicollinearity (Variance inflation factor, VIF > 2.5). Those that survived this step were included in a multivariable logistic regression with stepwise selection, assuming additivity and linearity. Eventually, stepwise binary logistic regression analysis was performed to identify the classifier variables for distinguishing people with PD from controls and freezers from non-freezers. The classifier variables were expressed as the odds ratio (OR) with 95% CI. This study considered two types of classification problems: PD classification (people with PD and controls) and FOG classification (Freezers and non-freezers). To resolve the classification problems, we introduced all 36 features from the 360° turning characteristics. We handled the features in two different ways to resolve the classification problems: (1) All 36 features were used for the PD and FOG classification problems; and (2) only features selected via stepwise regression as the feature selection approach were used. The five features selected for the PD classification problem were inner step length, step width, inner double support phase, thorax ROM, and incline angle. The six features selected for the FOG classification problem were outer step length, inner hip and ankle ROM, total distance of the COM, maximum anti-phase, and outer contralateral temporal coordination parameter. These features were selected using the aforementioned stepwise regression procedure. In addition, the optimal cut­off values of the turning features to identify people with PD and freezers with impairment in turning performance were identified using the receiver operating characteristic (ROC) curves. The Youden’s index (the highest sum of the values of sensitivity and specificity-1) was calculated to obtain the optimal cut­off values. Areas under the curve (AUC) of the ROC curves were calculated to measure the overall discriminative ability for people with PD and freezers. An AUC > 0.9 has high accuracy, whereas AUCs of 0.7–0.9 and 0.5–0.7 indicate moderate and low accuracies, respectively [36].

We used the 7 different ML approaches below for the classification problems (PD and FOG classification) to analyze the applicability of the proposed features from the 360° turning analysis to these problems. Generally, different machine learning algorithms gain different perspectives of the data when they are trained. These different perspectives cause variations in the accuracy of the classification problems. This variance should be evaluated and the basic approach is to use different ML algorithms to solve the classification problems. To solve the two classification problems, this study investigated seven traditional machine learning techniques: logistic regression (LR) [37], k-nearest neighbors (KNN) [38], naïve Bayes (NB) [39], linear discriminant analysis (LDA) [40], quadratic discriminant analysis (QDA) [40], support vector machine (SVM) [41], and random forest (RF) [42]. This study organized the results for the following 4 cases: (1) people with PD vs. controls with all thirty-six features (PD_36), (2) people with PD vs. controls with five features selected using stepwise regression (PD_5), (3) freezers vs. non-freezers with all thirty-six features (FOG_36), and (4) freezers vs. non-freezers with six features selected using stepwise regression (FOG_6). The model parameters of the classifiers were estimated using grid search. The estimated model parameters of the 4 cases are shown in Table 2. The accuracy, recall, precision, and F1 score were evaluated using fivefold cross validation in the analysis. Unfortunately, there was an imbalance in the collected patient dataset; the number of patients in the PD classification problem was 111 and the number of patients in the FOG classification problem was 77. Therefore, we handled these imbalanced samples using a random oversampling approach [43].

**Table 2:** Model parameters of the 7 classifiers estimated by grid search

ML techniques	PDs vs. Cons (with 36 features)	PDs vs. Cons (with 5 features)	F vs. NF (with 36 features)	F vs. NF (with 6 features)
LR	C = 1.0	C = 10.0	C = 0.1	C = 1000.0
KNN	k = 2	k = 4	k = 6	k = 2
NB	–	–	–	–
LDA	n_components = 1	n_components = 1	n_components = 1	n_components = 1
QDA	reg_param = 0.5	reg_param = 0.3	reg_param = 0.3	reg_param = 0.001
SVM	C = 29.6, gamma = 0.001, kernel = rbf	C = 7.6, gamma = 0.1, kernel = rbf	C = 7.6, gamma = 0.01, kernel = rbf	C = 1e-5, gamma = 10.0, kernel = rbf
RF	max_depth = 10, n_estimators = 500	max_depth = 20, n_estimators = 500	max_depth = 20, n_estimators = 1500	max_depth = 30, n_estimators = 500

*ML* machine learning, *PDs* people with PD, *Cons* controls, *F* people with PD with FOG, *NF* people with PD without FOG, *LR* logistic regression, “C” is the inverse of regularization strength, *KNN *k-nearest neighbors, “k” is the number of neighbors, *NB* Naïve Bayes, *LDA* linear discriminant analysis, “n_components” is the number of components, *QDA* quadratic discriminant analysis, “reg_param” is the regularization of the per-class covariance, *SVM* support vector machine, “C” is the regularization parameter and “gamma” is the kernel coefficient, *RF* random forest, “n_estimators” is the number of trees in the forest and “max_depth” is the maximum depth of the tree

In the multivariable linear regression analysis, to identify the independent associations between clinical and turning characteristics, each turning characteristic was applied to a multivariable linear regression model by using the stepwise regression method. Physical characteristics (age, gender, height, and BMI) were applied to the first block. Separate models were used for the turning characteristics; all variables were applied to the second block. The clinical characteristics were applied as the dependent variable. All statistical analyses were performed using SPSS 22.0 (SPSS Inc., Chicago, IL). The statistical significance level was set at 0.05.

## Results

### Classification using feature selection through stepwise regression

Table 3 shows the results of the stepwise regression procedure to select the features for classification of people with PD and controls, and freezers and non-freezers. In the classification of people with PD and controls (PD classification problem), the selected turning features were the inner step length (Cut­off value: 43.40 cm; AUC: 0.879, p < 0.001; sensitivity: 0.85; specificity: 0.81), step width (Cut­off value: 15.44 cm; AUC: 0.783, p < 0.001; sensitivity: 0.79; specificity: 0.79), inner double support phase (Cut­off value: 26.20%; AUC: 0.830, p < 0.001; sensitivity: 0.77; specificity: 0.77), Thorax ROM (Cut­off value: 33.72°; AUC: 0.629, p = 0.031; sensitivity: 0.68; specificity: 0.64), and incline angle (Cut­off value: 6.27°; AUC: 0.834, p < 0.001; sensitivity: 0.74; specificity: 0.73) during the 360° turning task. In the classification of freezers and non-freezers (FOG classification problem), the selected turning features were the outer step length (Cut­off value: 36.43 cm; AUC: 0.764, p < 0.001; sensitivity: 0.72; specificity: 0.68), inner hip ROM (Cut­off value: 31.11°; AUC: 0.632, p = 031; sensitivity: 0.61; specificity: 0.61), inner ankle ROM (Cut­off value: 19.73°; AUC: 0.517, p = 0.803; sensitivity: 0.50; specificity: 0.48), total distance of the COM (Cut­off value: 1.68 m; AUC: 0.531, p = 0.648; sensitivity: 0.57; specificity: 0.52), maximum anti-phase (Cut­off value: 17.89°; AUC: 0.636, p = 0.043; sensitivity: 0.61; specificity: 0.58), and outer contralateral temporal coordination parameter (Cut­off value: 0.77 s; AUC: 0.650, p = 0.026; sensitivity: 0.59; specificity: 0.58) during the 360° turning task.

**Table 3:** Feature selection of turning characteristics using stepwise regression procedure

Turning features (reference value)	OR (95% CI)	B (SE)	p-value	R_N_^2^
People with PD and controls				
Inner step length (43.40 cm)	0.709 (0.571–0.881)	− 0.343 (0.111)	**0.002**	0.896
Step width (15.44 cm)	1.548 (1.158–2.071)	0.437 (0.148)	**0.003**	
Inner double support phase (26.20%)	1.695 (1.134–2.533)	0.528 (0.205)	**0.010**	
Thorax ROM (33.72°)	1.048 (1.000–1.099)	0.047 (0.024)	**0.048**	
Incline angle (6.27°)	0.540 (0.326–0.895)	− 0.616 (0.258)	**0.017**	
Freezers and non-freezers				
Outer step length (36.43 cm)	0.720 (0.594–0.874)	− 0.328 (0.098)	**0.001**	0.650
Inner hip ROM (31.11°)	1.282 (1.087–1.512)	0.248 (0.084)	**0.003**	
Inner ankle ROM (19.73°)	0.823 (0.709–0.955)	− 0.195 (0.076)	**0.010**	
Total distance of the COM (1.68 m)	5.243 (1.093–25.148)	1.657 (0.800)	**0.038**	
Maximum anti-phase (17.89°)	0.827 (0.728–0.940)	− 0.189 (0.065)	**0.004**	
Outer contralateral temporal coordination (0.77 s)	30.958 (1.265–746.089)	3.433 (1.624)	**0.034**	

*PD* Parkinson’s disease, *OR* odds ratio, *B* logistic regression coefficient, *SE* standard error, *95% CI* 95% confidence interval, R_N_^2^ is the fit statistic for the Nagelkerke model, *ROM* range of motion, *COM* center of mass; adjusting for age, sex, height, and body mass index

Boldface indicates significant differences, p < 0.05

**Fig. 4:**
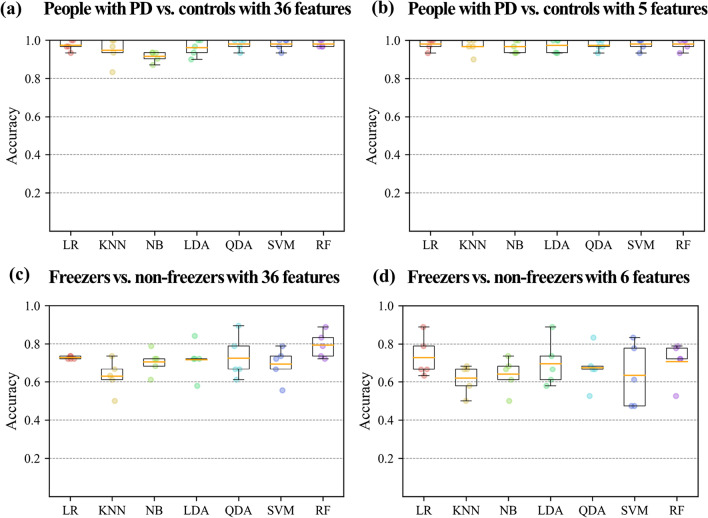
Accuracy of the 7 classifiers. The orange line in the box plot shows the mean values. *LR* logistic regression, *KNN* k-nearest neighbors, *NB* Naïve Bayes, *LDA* linear discriminant analysis, *QDA* quadratic discriminant analysis, *SVM* support vector machine, *RF* random forest

This study addressed two classification problems involving two different feature sets using all 360° turning features and features selected through stepwise regression. Table 4 indicates the average accuracy and SD of the classifiers, which was calculated through fivefold cross validation, and Fig. 4 shows the accuracy box plots of the PD and FOG classification problems in the 4 cases.

**Table 4:** Accuracies of 7 classifiers from fivefold cross validation

	ML techniques	PDs vs. Cons (with 36 features)	PDs vs. Cons (with 5 features)	F vs. NF (with 36 features)	F vs. NF (with 6 features)
Accuracy	LR	97.4 ± 2.8	**98.0 ± 3.0**	72.8 ± 0.8	**72.9 ± 10.8**
	KNN	94.7 ± 6.9	96.7 ± 4.1	62.9 ± 8.7	61.9 ± 7.8
	NB	91.6 ± 2.9*	96.7 ± 3.3*	70.6 ± 6.5	64.0 ± 9.0
	LDA	96.1 ± 4.3	97.4 ± 3.6	71.8 ± 9.3	69.6 ± 12.3
	QDA	98.0 ± 3.0	97.4 ± 2.8	72.6 ± 11.5	67.5 ± 10.9
	SVM	98.0 ± 3.0	**98.0 ± 3.0**	69.4 ± 8.9	63.4 ± 16.8
	RF	**98.1 ± 1.8**	**98.0 ± 3.0**	**79.4 ± 6.9**	70.8 ± 10.6

Mean (%) ± standard deviations (%) were calculated through fivefold cross validation; mean values presented in boldface denote the best performance (the highest test accuracy)

*ML* machine learning, *PDs* people with PD, *Cons* controls, *F* freezers, *NF* non-freezers, *LR* logistic regression, *KNN* k-nearest neighbors, *NB* Naïve Bayes, *LDA* linear discriminant analysis, *QDA* quadratic discriminant analysis, *SVM* support vector machine, *RF* random forest

*Denotes a significant difference

In the PD classification problem, naïve Bayes (NB) showed the lowest accuracy (91.6% ± 2.9% SD), and RF showed the highest accuracy (98.1% ± 1.8% SD) for PD_36. However, the difference in the accuracy of the classifiers was not large for PD_36, which means that most classifiers effectively distinguished between people with PD and controls. In addition, all classifiers showed similarly high accuracy performance after reducing the number of features. In this case, the LR, SVM, and RF showed the highest accuracy (98.0% ± 3.0% SD).

In the FOG classification problem, the k-nearest neighbors (KNN) had the lowest accuracy for FOG_36 (62.9% ± 8.7), whereas RF had the highest accuracy (79.4% ± 4.1%). Further, KNN had the lowest accuracy for FOG_6 (61.9% ± 7.8%), whereas LR had the highest accuracy (72.9% ± 10.8%). In the results of the paired t-test, there was no significant difference in the accuracies of LR (72.9% ± 10.8%) and RF (70.8% ± 10.6%): t(4) = 0.57, p = 0.60.

**Fig. 5:**
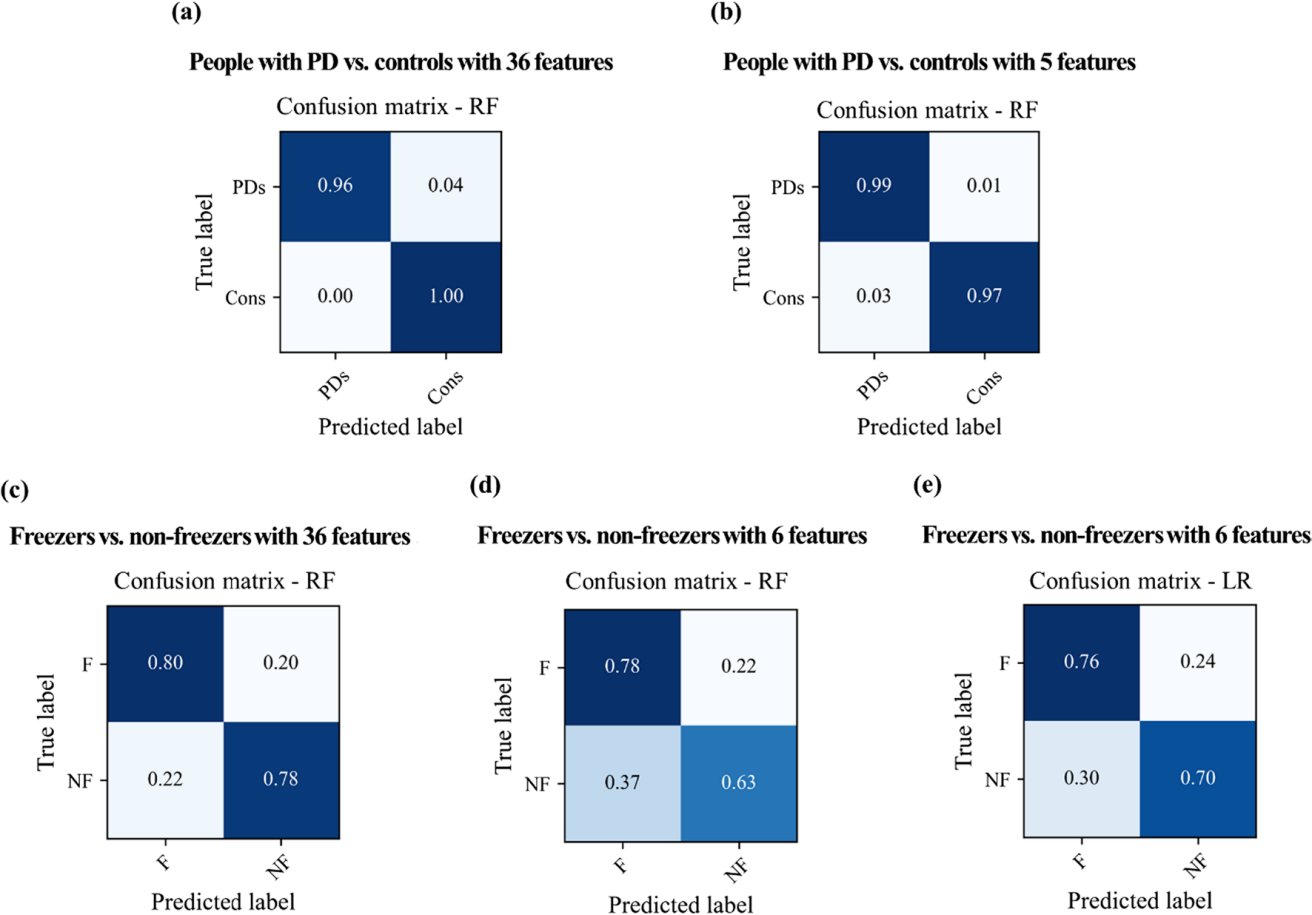
Confusion matrices of RF for the 4 cases and LR for FOG_6. *PDs* people with PD, *Cons* controls, *F* people with PD with FOG, *NF* people with PD without FOG, *RF* random forest, *LR* logistic regression

In addition, we investigated the confusion matrix to estimate the performance of the binary classification problems; the recall, precision, and F1 score results are shown in Table 5. Figure 5 shows the confusion matrices of RF in the 4 cases and LR in the FOG_6 case. In the PD classification problem, RF showed high accuracy performance, which was confirmed using the confusion matrix as well. There was no notable difference in the accuracy, recall, and precision between the confusion matrices of RF for PD_36 and PD_5. In the FOG classification problem, RF with the reduced feature set presented a performance degradation in identifying FOG when compared with RF with all 36 features; the true negative, which means the percentile of correctly identifying FOG, of RF degraded from 0.78 (percentile) for FOG_36 to 0.63 for FOG_6. LR showed a slightly higher accuracy than RF for FOG_6, but there was no significant difference. The comparison of the confusion matrices of RF and LR for FOG_6 showed a marginal difference; the true negative of RF was 0.63, and that of LR was 0.70. A statistically significant difference was not indicated, but we speculate that the correct identification of FOG is crucial to improve the performance of the FOG classification problem.

**Table 5:** Precision, recall, and F1 score of the 7 classifiers for 4 cases

	ML techniques	PDs vs. Cons (with 36 features)	PDs vs. Cons (with 5 features)	F vs. NF (with 36 features)	F vs. NF (with 6 features)
Precision	LR	97.6 ± 2.5	98.2 ± 2.6	74.2 ± 1.8	73.6 ± 10.7
	KNN	95.6 ± 5.3	97.1 ± 3.4	65.6 ± 9.7	67.4 ± 11.2
	NB	91.9 ± 3.1	97.1 ± 2.9	74.3 ± 4.0	65.1 ± 9.7
	LDA	96.3 ± 4.2	97.7 ± 3.2	72.6 ± 9.2	70.1 ± 12.1
	QDA	98.2 ± 2.6	97.6 ± 2.5	73.3 ± 11.5	68.2 ± 10.6
	SVM	98.2 ± 2.6	98.2 ± 2.6	72.1 ± 11.1	59.0 ± 33.6
	RF	98.2 ± 1.7	98.2 ± 2.6	81.4 ± 6.6	71.6 ± 10.3
Recall	LR	97.4 ± 2.8	98.0 ± 3.0	72.8 ± 0.8	72.9 ± 10.8
	KNN	94.7 ± 6.9	96.7 ± 4.1	62.9 ± 8.7	61.9 ± 7.8
	NB	91.6 ± 2.9	96.7 ± 3.3	70.6 ± 6.5	64.0 ± 9.0
	LDA	96.1 ± 4.3	97.4 ± 3.6	71.8 ± 9.3	69.6 ± 12.3
	QDA	98.0 ± 3.0	97.4 ± 2.8	72.6 ± 11.5	67.5 ± 10.9
	SVM	98.0 ± 3.0	98.0 ± 3.0	69.4 ± 8.9	63.4 ± 16.8
	RF	98.1 ± 1.8	98.0 ± 3.0	79.4 ± 6.9	70.8 ± 10.6
F1 score	LR	97.4 ± 2.8	98.0 ± 3.0	72.5 ± 0.9	72.7 ± 10.9
	KNN	94.6 ± 7.1	96.7 ± 4.1	61.6 ± 8.5	58.8 ± 8.7
	NB	91.6 ± 2.8	96.7 ± 3.3	69.1 ± 9.2	63.5 ± 9.0
	LDA	96.1 ± 4.3	97.4 ± 3.6	71.5 ± 9.4	69.5 ± 12.4
	QDA	98.0 ± 3.0	97.4 ± 2.8	72.4 ± 11.6	67.2 ± 11.2
	SVM	98.0 ± 3.0	98.0 ± 3.0	68.8 ± 8.8	54.9 ± 24.7
	RF	98.1 ± 1.8	98.0 ± 3.0	79.1 ± 7.1	70.4 ± 10.9

Precision, recall, and F1 score are represented as mean (%) ± standard deviation (%)

*ML* machine learning, *PDs* people with PD, *Cons* controls, *F* people with PD with FOG, *NF* people with PD without FOG, *LR* logistic regression, *KNN* k-nearest neighbors, *NB* Naïve Bayes, *LDA* linear discriminant analysis, *QDA* quadratic discriminant analysis, *SVM* support vector machine, *RF* random forest

### Association between clinical and 360° turning characteristics in people with PD

To investigate the association between the clinical and 360° turning characteristics, we used 5 selected features in the PD classification and 6 selected features in the FOG classification, which are extracted via stepwise regression. The next step was to further analyze the associations between the clinical and turning features selected using a stepwise linear regression model after adjusting for age, sex, height, and BMI.

**Fig. 6:**
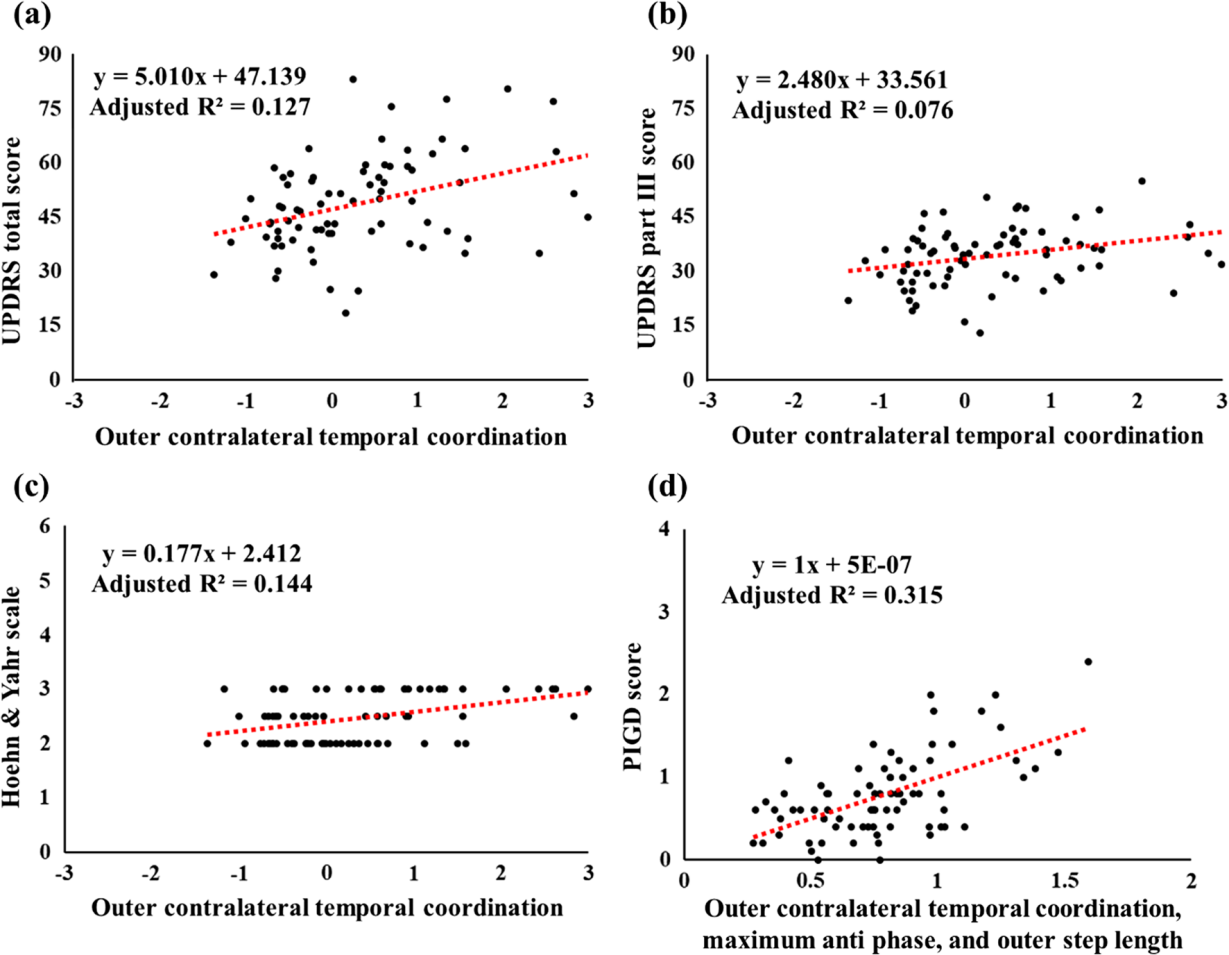
Stepwise multivariable linear regression models for associations clinical and turning characteristics of people with PD. (**a**) As the UPDRS total score, (**b**) UPDRS part III score, and (**c**) Hoehn and Yahr stage increases, the outer contralateral temporal coordination parameter increases; (**d**) as the PIGD score increases, the outer contralateral temporal coordination parameter, maximum anti-phase, and outer step length increases for people with PD when performing the 360° turning task. The tendencies of the regression lines indicate the positive or negative linear correlation of their corresponding correlation coefficients. Significant differences are indicated by p < 0.05; *UPDRS* Unified Parkinson’s Disease Rating Scale, *PIGD* Postural instability/gait difficulty

The results of the linear regression model for people with PD showed that as the UPDRS total score (p = 0.001) (Fig. 6a), UPDRS part III score (p = 0.009) (Fig. 6b), and Hoehn and Yahr stage (p < 0.001) (Fig. 6c) increases, the outer contralateral temporal coordination parameter increases for people with PD when performing the 360° turning task. In addition, as the Postural Instability/Gait Difficulty (PIGD) score (p < 0.001) (Fig. 6d) increases, they show an increase in the outer contralateral temporal coordination parameter, maximum anti-phase, and outer step length.

**Fig. 7:**
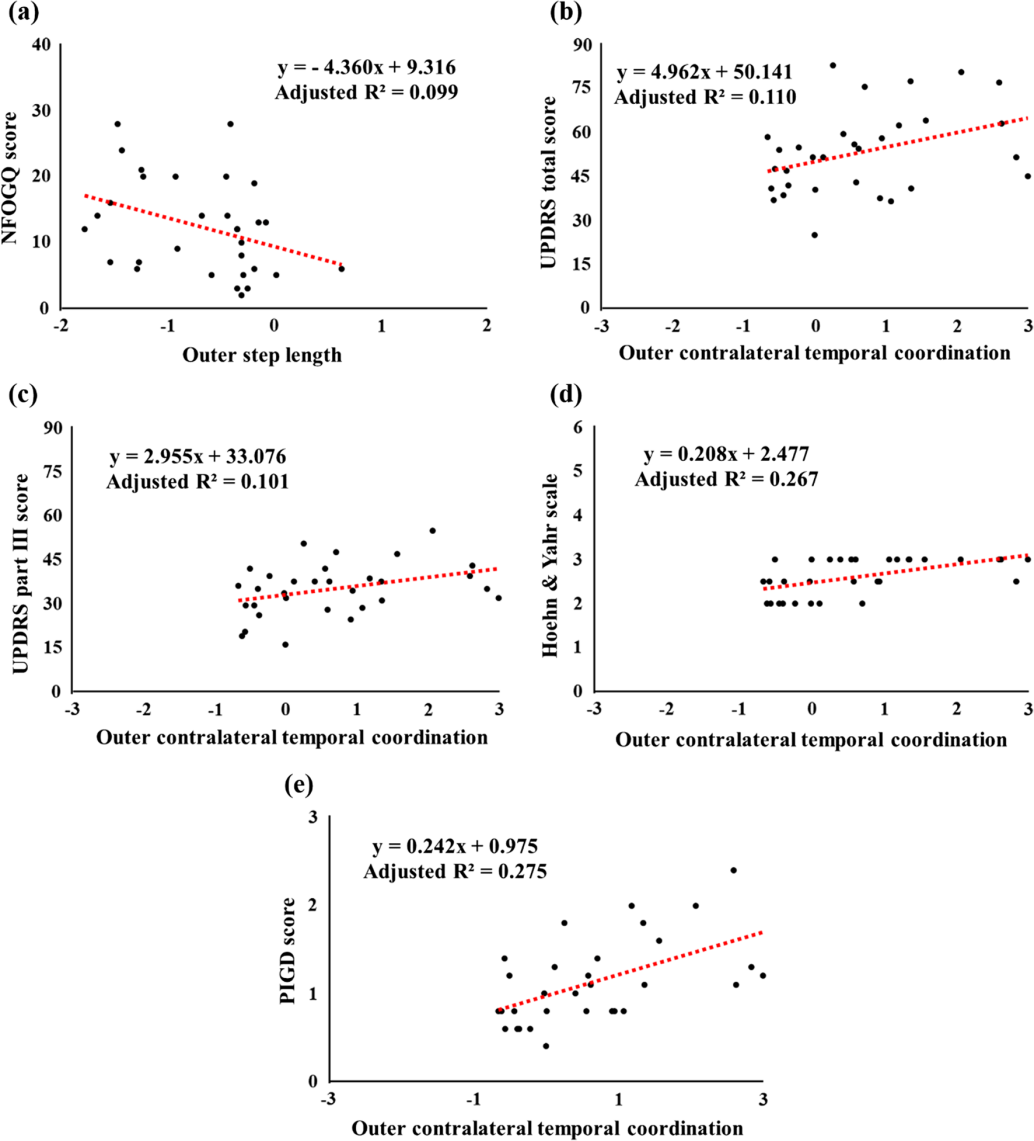
Stepwise multivariable linear regression models for associations clinical and turning characteristics of freezers. (**a**) As the NFOGQ score increases, the outer step length decrease; (**b**) as the UPDRS total score, (**c**) UPDRS part III score, (**d**) Hoehn and Yahr stage, and (**e**) PIGD score increase, the outer contralateral temporal coordination parameter increases for freezers when performing the 360° turning task. The tendencies of the regression lines indicate the positive or negative linear correlation of their corresponding correlation coefficients. Significant differences are indicated by p < 0.05; *NFOGQ* New freezing of gait questionnaire, *UPDRS* Unified Parkinson’s Disease Rating Scale, *PIGD* Postural instability/gait difficulty

The results of the linear regression model for freezers revealed that as the NFOGQ score (p = 0.047) (Fig. 7a) increases, freezers show a decrease in the outer step length. In addition, as the UPDRS total score (p = 0.038) (Fig. 7b), UPDRS part III score (p = 0.045) (Fig. 7c), Hoehn and Yahr stage (p = 0.002) (Fig. 7d), and PIGD score (p = 0.001) (Fig. 7e) increase, they show an increase in the outer contralateral temporal coordination parameter.

Additionally, we evaluated the Spearman’s rank correlation to investigate the relationship between the clinical characteristics, disease duration, and levodopa equivalent dose. The NFOGQ score (r_s_ = 0.405, p < 0.001), UPDRS total score (r = 0.325, p = 0.004; Pearson correlation), PIGD score (r_s_ = 0.358, p = 0.001), and levodopa equivalent dose (r_s_ = 0.521, p < 0.001) were strongly correlated with the disease duration.

## Discussion

In the main findings for people with PD and controls of this study, the five selected features that were most relevant for the classification of people with PD and controls were the inner step length, step width, inner double support phase, thorax ROM, and incline angle during the 360° turning task. The machine learning approach showed that RF solved the PD classification problem with 98.1% and 98.0% accuracies for PD_36 and PD_5, respectively. In the main findings for freezers and non-freezers, the six selected features that were most relevant for the classification of freezers and non-freezers were outer step length, inner hip and ankle ROM, total distance of the COM, maximum anti-phase, and outer contralateral temporal coordination parameter during the 360° turning task. The machine learning approach showed that RF had 79.4% accuracy for FOG_36 and LR had 72.9% accuracy for FOG_6. Additionally, the 360° turning characteristics such as outer contralateral temporal coordination parameter, maximum anti-phase, and outer step length were associated with the clinical characteristics of people with PD and freezers. Therefore, the 360° turning features based on full-body kinematic analysis may enable classification of people with PD and controls, freezers and non-freezers, and its association with clinical characteristics is demonstrated. These findings are discussed in detail below.

### Classification using feature selection through stepwise regression

In our study on the classification of people with PD and controls, 5 features were selected through stepwise regression to obtain the sensitive cut­off values in the ROC analysis. These turning features are related to the spatiotemporal parameters and turning strategy for the inner step of the more affected limb. People with PD showed a significantly shorter step length, wider step width, longer double support phase, greater thorax ROM, and smaller incline angle for maintaining their center of gravity between the two feet when compared to those with controls [44]. The supplementary motor area, which receives input from the impaired basal ganglia in people with PD, participates in the control of postural coordination and affects the bilateral function of gait [45]. These results may cause dynamic instability during turning because people with PD present a lower supplementary motor area activity than with controls [46]. Therefore, we suggest that people with PD and controls may be distinguished using turning features such as spatiotemporal parameters, trunk ROM, and incline angle related to coupling between posture and gait during turning tasks for the inner step of the more affected limb [47].

The machine learning approach showed that RF resolved the PD classification problem with 98.1% and 98.0% accuracies for PD_36 and PD_5, respectively. From these results, the possibility of distinguishing between people with PD and controls based on the 360° turning characteristics was confirmed to some extent. In the PD classification problem, the feature selection approach by stepwise regression showed reasonable accuracy performance. RF outperformed all other classifiers with all 36 features; in addition, LR, SVM, and RF with the reduced feature set performed better than the other classifiers in resolving the classification problems. These results indicated that feature selection by stepwise regression removed irrelevant features. Generally, the output of a model can be affected by multiple features. When the number of features increases, the model becomes complicated. An overfitting model tends to consider all features, even though some of them have very limited effect on the final output [48].

For classification of freezers (disease duration: 8.39 ± 5.83 years) and non-freezers (disease duration: 4.36 ± 3.61 years), six features were selected via stepwise regression to obtain the sensitive cut­off values in the ROC analysis. These turning features are related to the turning strategy and interlimb coordination. Freezers showed a significantly shorter step length, greater hip ROM, smaller ankle ROM, longer total distance of the COM, smaller maximum anti-phase, and longer contralateral temporal coordination parameter using the compensatory strategy for postural instability when compared with those of non-freezers. In particular, to observe a phase delay between the upper and lower limbs in people with PD and freezers, temporal coordination while turning may be used as the primary parameter. During turning, delayed temporal coordination between the upper and lower limbs indicates a reduced coordination capacity [49, 50]. In addition, our result showed that freezers have a dependent turning characteristic by shortening the outer step length of the rotation center, along with en bloc head and trunk rotation compared to non-freezers [51]. These characteristics may increase the risk of falls owing to potential FOG characteristics, suggesting that they may experience greater turning difficulty due to increased postural instability with disease progression [52]. It may be caused by deficits in several components of postural control, such as anticipatory postural adjustments, delayed reaction time, abnormal automatic postural reactions, and abnormal axial kinesthesia [53]. The turning task threatens the stability of freezers more than any other freezing trigger as it requires a precise control of each limb [26]. In addition, freezers showed less rhythmic and uncoordinated gait patterns when compared to those of non-freezers [45]. These results suggest that freezers may experience difficulties in performing automatized movements without adequate attention [54] and may be more vulnerable to impairments related to interlimb coordination because turning is asymmetrical when compared with a straight gait [55]. Therefore, we suggest that freezers and non-freezers can be classified based on the turning features related to postural transitions and coordination [56].

The machine learning approach showed that RF resolved the FOG classification problem with 79.4% accuracy for FOG_36, and LR resolved it with 72.9% accuracy for FOG_6. From the results, the possibility of distinguishing between freezers and non-freezers based on the 360° turning characteristics was confirmed to some extent; however, the FOG classification problem appears more challenging than the PD classification problem. First, no classifier had high accuracy of more than 80%. Moreover, the SD of the accuracy for FOG_6 was higher for all classifiers except KNN and QDA when compared with the results for FOG_36; especially, the SDs of the accuracies of SVM and LR showed a rapid increase (the value for SVM ranged from 8.9 to 16.8% whereas that for LR ranged from 0.8 to 10.8%). We speculate that this was caused by the relatively small sample size in this study. The small number of samples might cause a misinterpretation in the mathematical optimization procedure while the classifier is being trained, and it might affect the performance of SVM and LR because of the nature of these classification algorithms. In future research to improve the accuracy of the FOG classification problem, the raw time series motion data during the 360° turning task need to be studied via advanced deep learning techniques such as the n-dimensional convolutional neural network and recurrent neural network. Although the raw motion data are converted to selected 36 features, there is a possibility of losing key information required to solve the FOG classification problem.

### Associations between clinical and 360° turning characteristics of people with PD

This study conducted feature selection using stepwise regression for the 360° turning characteristics. Based on the selected turning characteristics, the associations between the clinical and turning characteristics of people with PD and freezers were investigated. We observed the associations between the clinical characteristics such as the UPDRS total and UPDRS III scores, Hoehn and Yahr stage, PIGD score, and NFOGQ score, and the selected features during the 360° turning task. Although our result was similar to the findings of the previous studies on the associations between the severity of PD and turning characteristics [57–60], most studies employed small sample sizes and often did not control for confounders that may affect the turning characteristics owing to physical characteristics such as age, sex, height, and BMI. In addition, the previous studies assessed the clinical characteristics in the “On” state of medication [58, 60], whereas this study assessed the clinical characteristics and turning task of people with PD in the “Off” state of medication. The medication status of people with PD influences the motor symptoms and may affect the generalization limitations of the associations between clinical and turning characteristics of people with PD who exhibit FOG [61, 62]. A previous study reported that people with PD and freezers showed a more constrained movement during turning in the “Off” state of medication when compared with the controls and non-freezers [63]. The study considered a compensation strategy for preventing falls in people with PD and freezers, which were caused by the declined ability to control the centrifugal forces that create the inertia forces to allow body rotation, especially immediately after the pivot point during turning [7]. In addition, as dynamic stability is already compromised in people with PD and freezers, they may have shown more careful movement during the turning [64, 65]. A more constrained postural strategy may be used to facilitate effective turning under dopamine depletion, which may influence the control of automatized movement [63, 66]. Especially, freezers need a strategy to increase their stability during turning owing to greater impairment of cognitive, executive, and attentional resources when compared with non-freezers [20, 67, 68].

We showed that PD severity for motor symptoms is related to a decrease in turning performance. Turning is an asymmetric task, in which one limb generates a stepping pattern, and the other helps with weight shifting and support; thus, it requires a higher level of bilateral coordination in people with PD and freezers [45]. In a majority of such people, the right limb is initially affected to a greater extent, suggesting a decline in the neural control ability during turning due to certain associations between the symptom-dominant side and dominant hemisphere [45]. In addition, a higher PIGD score was significantly associated with greater maximum anti-phase and shorter outer step length while turning in people with PD. This result showed that the severity of axial symptoms and gait difficulties during turning, and not the general severity of PD, might affect the turning performance [57]. Our study using the 360° turning task for the inner step of the more affected limb identified association with clinical characteristics of people with PD and freezers through the difference in turning characteristics according to disease severity for motor symptoms. Previous studies have reported that the more affected limb of people with PD tends to be affected predominantly throughout disease progression and may promote greater motor deficits [16, 69]. This suggests that the turning difficulty may be a result of asymmetry between the more and less affected limbs and impaired in both automatic and controlled processes [9, 70]. Therefore, we suggest that a more challenging 360° turning task for the inner step of the more affected limb may be evaluated through the turning performance of people with PD and freezers.

Furthermore, clinical characteristics related to PD severity, such as UPDRS total and III scores, PIGD score, Hoehn and Yahr stage, and NFOGQ score, were identified as the indicators of FOG [71]. Previous studies have shown the association of the severity of FOG with motor deficit [72, 73]. It has been suggested that induced motor deficit such as the loss of automaticity along with stepping inhibition during turning led to repeated weight shifts without stepping, resulting in trembling of limbs related to FOG [71, 72]. In particular, our result indicated that the outer step length decreased as the NFOGQ score increased in freezers. In this study, no difference between the inner and outer step lengths in freezers was observed during the turning task. These results do not indicate the asymmetry of steps during turning in freezers with advanced disease severity [74, 75], which may be reflected as reduced normal asymmetric gait strategy and bilateral motor coordination during turning [74].

Additionally, we observed the correlation of the NFOGQ, UPDRS total, PIGD score, and levodopa equivalent dose with disease duration. The advanced severity and long duration of the disease along with disease progression in people with PD may contribute to the severity of FOG [75]. There was also a significant correlation between the PIGD score and disease duration, which could lead to axial symptoms including gait disturbance and postural abnormalities in freezers with longer disease duration when compared with non-freezers [76]. Although people with PD are likely to develop FOG over time (it may be noted that all people with PD do not develop FOG), other factors such as the disease duration and dopaminergic treatment as well as genetic status may also influence gait disturbance [77].

Our study had several limitations. First, the effects of the “On” and “Off” states of medication and the differences in the turning direction were not compared while evaluating the 360° turning tasks. Second, our datasets have an imbalance related to gender and use different sample sizes. The results are expected to improve with a more homogeneous dataset. However, we analyzed after adjusting for the covariates of age, sex, height, and BMI. Third, the sample size of freezers in the FOG classification problem was relatively small: 34 freezers and 43 non-freezers. Although we used the random oversampling technique to handle this imbalanced dataset, the inadequate sample size was likely to cause instability of the classification performance of SVM and LR, as mentioned previously. In addition, a FOG episode was induced in one participant during turning for the inner step of the more affected limb; the corresponding results were excluded from the analysis. Fourth, the R^2^ values for the associations between disease severity and turning characteristics of people with PD are weak. Thus, further study using instruments to assess various clinical characteristics in the medication “On” and “Off” states and longitudinal studies are needed to generalize the associations between disease severity based on the clinical characteristics and the turning characteristics. Fifth, for previous studies, many measures related to disease severity (duration of disease, UPDRS total and III scores, and levodopa equivalent dose) of people with PD have been significantly different between freezers and non-freezers [31, 71, 72, 76]. However, our result that no difference in UPDRS III between freezers and non-freezers (p = 0.565). Research suggested that although UPDRS III may contribute to assessing the functional impact of FOG, there do not reflect the overall severity of FOG [31]. Therefore, further research is needed considering the sample size and objective evaluation status of freezers and non-freezers. Lastly, machine learning techniques with higher predictability for classification and a filtering technique for motor symptoms of people with PD and freezers need to be developed. A method employing a larger sample size or important factors contributing to improving the evaluation of disease severity and predictability of classification and diagnosis may be added to the classification model. Further studies are needed to evaluate the realistic patient movements on the raw time series motion data through advanced machine learning techniques such as deep learning.

The findings of this study have some important implications. First, the results of the turning characteristics for the inner step of the more affected limb in people with PD and freezers may be helpful in improving the clinical assessment and understanding of disease severity by disease progression. Second, the machine learning approach to resolve the PD and FOG classification problems of this study showed similar results when using kinematic features selected through 360° turning analysis. This result may be helpful in understanding the movement characteristics and classifying the disease severity of people with PD and freezers based on certain main factors of the spatiotemporal and kinematic features during turning tasks. Third, the clinical characteristics were shown to be associated with the turning characteristics. These results may help in ameliorating the motor symptoms of people with PD and improving the rehabilitative strategies, which may reduce the occurrence of freezing.

## Conclusion

Feature selection through stepwise regression was used to select the meaningful turning features for the classification of people with PD and controls and freezers and non-freezers. The next step based on the machine learning approach showed similar results wherein RF showed the highest classification accuracy of 98.1% in the PD classification problem when using all 360° turning features, and 98.0% when using the five selected features; RF and LR showed 79.4% accuracy in the FOG classification problem when using all 360° turning features, and 72.9% accuracy when using the six selected features. In addition, the results for the associations between the clinical and turning characteristics showed that lower turning performance might indicate increased disease severity. We suggest that our results understand the turning characteristics of people with PD and freezers during the 360° turning task for the inner step of the more affected limb and may help in improving the objective classification and clinical assessment by disease progression using turning features selected. Further in-depth studies based on machine learning are required as turning factors may support the classification of PD, and changes in severity of motor symptoms can be assessed through sensor-based motion analysis in daily life.

## Supplementary Information

**Additional file 1: Table S1.** Turning characteristics of PD patients in comparison with controls, and freezers in comparison with non-freezers, during 360° turning task.

## Data Availability

The datasets that support the findings of this study are available from the corresponding author upon reasonable request.

## Abbreviations

PD: Parkinson’s disease
FOG: Freezing of gait
Freezers: People with PD with FOG
Non-freezers: People with PD without FOG
IMA: Inner step of the more affected limb
OMA: Outer step of the more affected limb
AP: Anteroposterior
ML: Mediolateral
RMS: Root mean square
COM: Center of mass
MMSE: Mini mental state examination
NFOGQ: New freezing of gait questionnaire
IRB: Institutional review board
3D: Three-dimensional
UPDRS: Unified Parkinson disease rating scale
CI: Confidence interval
SD: Standard deviations
ANOVA: Analysis of variance
ES: Effect size
OR: Odds ratios
ANCOVA: Analysis of covariance
BMI: Body mass index
VIF: Variance inflation factor
ROC: Receiver operating characteristic
AUC: Areas under the curve
LR: Logistic regression
KNN: K-nearest neighbors
NB: Naїve Bayes
LDA: Linear discriminant analysis
QDA: Quadratic discriminant analysis
SVM: Support vector machine
RF: Random forest
PD_36: People with PD vs. controls with thirty-six features
PD_5: People with PD vs. controls with five features selected using stepwise regression
FOG_36: Freezers vs. non-freezers with thirty-six features
FOG_6: Freezers vs. non-freezers with six features selected by stepwise regression
PIGD: Postural instability/gait difficulty
