# Supplementary material for: Classification of Parkinson’s disease with freezing of gait based on 360° turning analysis using 36 kinematic features
Source: J Neuroeng Rehabil. 2021 Dec 20;18:177. doi: 10.1186/s12984-021-00975-4 (PMC8686361; doi:10.1186/s12984-021-00975-4)
Supplement: Supplementary file 1 — Additional file 1: Table S1. Turning characteristics of PD patients in comparison with controls, and freezers in comparison with non-freezers, during 360° turning task. [file 12984_2021_975_MOESM1_ESM.docx]

| **Table S1.** Turning characteristics of PD patients in comparison with controls, and freezers in comparison with non-freezers, during 360° turning task. | | | | | | | | |  |  |
| --- | --- | --- | --- | --- | --- | --- | --- | --- | --- | --- |
| **Turning characteristics** | **PD patients**  **(95% CI)** | **Controls**  **(95% CI)** | **p^a^**  **value** | **ES** | **Freezers**  **(95% CI)** | **Non-freezers**  **(95% CI)** | **p^b^**  **value** | **ES** | |  |
| Total steps | 9.17 ± 0.03  (8.50–9.87) | 8.89 ± 3.15  (7.79–10.07) | 0.716 | 0.00 | 10.02 ± 3.08  (9.00–11.22) | 8.59 ± 2.89  (7.76–9.40) | **0.033** | 0.06 | |  |
| Total duration (s) | 4.95 ± 2.22  (4.45–5.46) | 4.66 ± 2.11  (3.97–5.37) | 0.649 | 0.00 | 5.00 ± 1.92  (4.39–5.76) | 4.91 ± 2.41  (4.20–5.63) | 0.585 | 0.00 | |  |
| Step width (cm) | 18.85 ± 5.45  (17.64–20.13) | 13.16 ± 5.05  (11.60–14.91) | **<0.001** | 0.20 | 17.24 ± 5.18  (15.33–19.12) | 19.94 ± 5.41  (18.18–21.41) | **0.036** | 0.06 | |  |
| Inner step length (cm) | 36.47 ± 8.30  (34.69–38.21) | 48.55 ± 5.29  (46.93–50.38) | **<0.001** | 0.36 | 32.15 ± 6.16  (30.12–34.40) | 39.37 ± 8.35  (36.93–41.75) | **<0.001** | 0.17 | |  |
| Outer step length (cm) | 37.03 ± 8.25  (35.24–38.86) | 48.66 ± 6.15  (46.63–50.82) | **<0.001** | 0.34 | 32.48 ± 6.09  (30.25–34.59) | 40.09 ± 8.14  (37.67–42.26) | **<0.001** | 0.18 | |  |
| Inner single support phase (%) | 37.99 ± 2.90  (37.42–38.65) | 38.80 ± 3.19  (37.70–39.92) | 0.244 | 0.01 | 38.68 ± 3.35  (37.50–39.89) | 37.52 ± 2.48  (36.89–38.25) | 0.111 | 0.04 | |  |
| Outer single support phase (%) | 37.97 ± 3.24  (37.29–38.75) | 37.92 ± 2.68  (37.06–38.82) | 0.959 | 0.00 | 38.54 ± 3.10  (37.53–39.58) | 37.58 ± 3.31  (36.66–38.57) | 0.234 | 0.02 | |  |
| Inner double support phase (%) | 28.28 ± 4.14  (27.33–29.12) | 23.58 ± 3.02  (22.55–24.52) | **<0.001** | 0.28 | 28.65 ± 3.58  (27.32–29.84) | 28.02 ± 4.50  (26.71–29.25) | 0.197 | 0.02 | |  |
| Outer double support phase (%) | 27.70 ± 4.21  (26.78–28.51) | 23.50 ± 3.10  (22.42–24.46) | **<0.001** | 0.23 | 28.16 ± 3.74  (26.83–29.40) | 27.39 ± 4.50  (25.99–28.75) | 0.116 | 0.03 | |  |
| Inner stance phase (%) | 66.20 ± 4.01  (65.28–67.10) | 62.34 ± 2.50  (61.46–63.16) | **<0.001** | 0.22 | 67.33 ± 3.77  (65.97–68.62) | 65.43 ± 4.03  (64.24–66.60) | **0.006** | 0.10 | |  |
| Outer stance phase (%) | 65.57 ± 3.70  (64.81–66.43) | 61.42 ± 2.77  (60.47–62.25) | **<0.001** | 0.25 | 66.71 ± 4.14  (65.18–68.20) | 64.80 ± 3.19  (63.85–65.81) | **0.002** | 0.13 | |  |
| Inner hip ROM (°) | 30.86 ± 7.85  (29.06–32.55) | 37.86 ± 8.84  (34.83–40.87) | **<0.001** | 0.14 | 33.12 ± 7.62  (30.48–35.85) | 29.34 ± 7.71  (27.17–31.67) | 0.063 | 0.05 | |  |
| Outer hip ROM (°) | 32.16 ± 7.32  (30.53–33.72) | 34.11 ± 10.28  (30.74–37.66) | 0.227 | 0.01 | 32.90 ± 7.05  (30.45–35.49) | 31.66 ± 7.54  (29.63–34.01) | 0.473 | 0.01 | |  |
| Inner knee ROM (°) | 46.21 ± 11.21  (43.69–48.60) | 49.38 ± 11.94  (45.44–53.52) | 0.269 | 0.01 | 47.04 ± 9.52  (43.84–50.35) | 45.65 ± 12.29  (42.04–49.74) | 0.879 | 0.00 | |  |
| Outer knee ROM (°) | 46.35 ± 13.36  (43.42–49.59) | 50.38 ± 12.63  (46.44–54.62) | 0.140 | 0.02 | 49.08 ± 15.51  (44.44–55.10) | 44.51 ± 11.52  (41.33–47.99) | 0.416 | 0.01 | |  |
| Inner ankle ROM (°) | 21.50 ± 7.57  (19.88–23.32) | 32.73 ± 12.30  (28.42–36.76) | **<0.001** | 0.23 | 21.09 ± 5.75  (19.29–23.11) | 21.78 ± 8.63  (19.50–24.39) | 0.651 | 0.00 | |  |
| Outer ankle ROM (°) | 22.44 ± 8.43  (20.80–24.62) | 34.89 ± 11.83  (30.79–38.97) | **<0.001** | 0.25 | 23.03 ± 9.88  (19.99–26.93) | 22.04 ± 7.40  (20.01–24.35) | 0.794 | 0.00 | |  |
| Inner toe clearance height (cm) | 6.66 ± 1.27  (6.37–6.95) | 7.22 ± 1.39  (6.79–7.68) | 0.071 | 0.03 | 6.61 ± 1.44  (6.12–7.13) | 6.70 ± 1.15  (6.37–7.06) | 0.327 | 0.01 | |  |
| Outer toe clearance height (cm) | 6.27 ± 1.19  (6.02–6.52) | 7.11 ± 1.52  (6.64–7.68) | **0.003** | 0.08 | 6.37 ± 1.40  (5.87–6.88) | 6.20 ± 1.04  (5.92–6.52) | 0.911 | 0.00 | |  |
| Inner shoulder ROM (°) | 20.13 ± 11.78  (17.78–22.77) | 31.14 ± 14.62  (26.20–35.99) | **<0.001** | 0.13 | 19.55 ± 11.07  (15.90–23.36) | 20.53 ± 12.35  (17.19–24.29) | 0.533 | 0.01 | |  |
| Outer shoulder ROM (°) | 20.54 ± 12.89  (17.88–23.44) | 27.53 ± 17.74  (22.25–34.06) | **0.034** | 0.04 | 22.53 ± 12.51  (18.04–27.31) | 19.21 ± 13.10  (15.57–23.36) | 0.576 | 0.00 | |  |
| Pelvis ROM (°) | 51.68 ± 27.72  (45.89–57.96) | 37.71 ± 28.54  (28.65–47.17) | **0.019** | 0.05 | 46.49 ± 28.00  (37.24–56.55) | 55.19 ± 27.28  (47.43–62.80) | 0.229 | 0.02 | |  |
| Thorax ROM (°) | 50.68 ± 28.61  (44.66–56.78) | 37.62 ± 27.74  (28.99–47.52) | **0.031** | 0.04 | 45.22 ± 27.56  (36.21–54.74) | 54.36 ± 29.02  (46.26–62.52) | 0.175 | 0.026 | |  |
| Thorax forward ROM (°) | 10.77 ± 8.25  (9.00–12.27) | 7.62 ± 4.25  (5.45–10.41) | 0.076 | 0.03 | 9.79 ± 7.40  (6.90–12.685) | 11.44 ± 8.79  (9.08–13.80) | 0.390 | 0.01 | |  |
| Thorax lateral ROM (°) | 3.35 ± 2.55  (2.83–3.92) | 2.71 ± 2.13  (1.82–3.49) | 0.158 | 0.02 | 3.16 ± 2.49  (2.31–4.17) | 3.47 ± 2.61  (2.66–4.18) | 0.761 | 0.00 | |  |
| Maximum anti phase (°) | 19.99 ± 7.72  (18.41–21.75) | 18.02 ± 4.85  (16.53–19.57) | 0.262 | 0.01 | 17.98 ± 8.18  (15.05–20.98) | 21.35 ± 7.17  (19.26–23.62) | 0.143 | 0.03 | |  |
| Incline angle (°) | 4.92 ± 1.87  (4.51–5.33) | 8.42 ± 3.02  (7.46–9.44) | **<0.001** | 0.33 | 4.72 ± 1.69  (4.18–5.34) | 5.05 ± 1.98  (4.49–5.63) | 0.385 | 0.01 | |  |
| Inner ipsilateral tempo (s) | 0.22 ± 0.09  (0.20–0.24) | 0.16 ± 0.07  (0.14–0.19) | **0.002** | 0.09 | 0.23 ± 0.10  (0.19–0.26) | 0.22 ± 0.08  (0.19–0.24) | 0.349 | 0.01 | |  |
| Outer ipsilateral tempo (s) | 0.24 ± 0.10  (0.22–0.26) | 0.15 ± 0.07  (0.12–0.17) | **<0.001** | 0.20 | 0.28 ± 0.10  (0.25–0.31) | 0.22 ± 0.09  (0.19–0.24) | **0.003** | 0.12 | |  |
| Inner contralateral tempo (s) | 0.77 ± 0.29  (0.71–0.84) | 0.52 ± 0.18  (0.46–0.57) | **<0.001** | 0.18 | 0.84 ± 0.34  (0.72–0.97) | 0.73 ± 0.24  (0.66–0.80) | **0.021** | 0.07 | |  |
| Outer contralateral tempo (s) | 0.80 ± 0.29  (0.73–0.86) | 0.49 ± 0.18  (0.43–0.55) | **<0.001** | 0.24 | 0.90 ± 0.32  (0.78–1.01) | 0.73 ± 0.25  (0.66–0.80) | **0.004** | 0.11 | |  |
| Area of the COM (m^2^) | 2.48 ± 0.50  (2.37–2.60) | 2.41 ± 0.58  (2.22–2.61) | 0.687 | 0.00 | 2.46 ± 0.48  (2.31–2.65) | 2.49 ± 0.52  (2.35–2.64) | 0.825 | 0.00 | |  |
| AP RMS distance of the COM (m) | 0.19 ± 0.04  (0.19–0.20) | 0.20 ± 0.06  (0.18–0.22) | 0.642 | 0.00 | 0.20 ± 0.05  (0.19–0.22) | 0.19 ± 0.03  (0.18–0.20) | 0.231 | 0.02 | |  |
| ML RMS distance of the COM (m) | 0.23 ± 0.11  (0.21–0.26) | 0.26 ± 0.19  (0.21–0.33) | 0.199 | 0.02 | 0.22 ± 0.08  (0.20–0.25) | 0.24 ± 0.13  (0.20–0.28) | 0.878 | 0.00 | |  |
| Total distance of the COM (m) | 1.79 ± 0.59  (1.65–1.93) | 1.70 ± 0.71  (1.46–1.97) | 0.914 | 0.00 | 1.82 ± 0.59  (1.62–2.02) | 1.77 ± 0.60  (1.61–1.95) | 0.461 | 0.01 | |  |
| Total velocity of the COM (m/s) | 0.48 ± 0.22  (0.43–0.53) | 0.42 ± 0.24  (0.34–0.49) | 0.184 | 0.02 | 0.49 ± 0.22  (0.40–0.55) | 0.47 ± 0.22  (0.42–0.54) | 0.968 | 0.00 | |  |
| All data are mean ± standard deviations (95% confidence interval); p^a^-value: analysis of covariance (ANCOVA) between PD patients and controls; p^b^-value: ANCOVA between freezers and non-freezers; ES: effect size; ROM: range of motion; Tempo: temporal coordination of the upper and lower limbs; AP: anteroposterior; ML: mediolateral; COM: center of mass; Adjustment for age, sex, height, and body mass index; Boldface indicates significant differences, p < 0.05. | | | | | | | | | | |
